# Physical Characteristics of Medical Textile Prostheses Designed for Hernia Repair: A Comprehensive Analysis of Select Commercial Devices

**DOI:** 10.3390/ma8125453

**Published:** 2015-12-02

**Authors:** Linli Miao, Fang Wang, Lu Wang, Ting Zou, Gaétan Brochu, Robert Guidoin

**Affiliations:** 1 Key laboratory of Textile Science and Technology of Ministry of Education, College of Textiles, Donghua University, Songjiang District, Shanghai 201600, China; miaolinli_1990@126.com (L.M.); wangfang916@126.com (F.W.); zoutingdhu@gmail.com (T.Z.); robertguidoin@hotmail.com (R.G.); 2 Department of Surgery, Université Laval and Research Center, Centre hospitalier universitaire de Québec, Québec, QC G1V 0A6, Canada; Gaetan.Brochu@fmed.ulaval.ca

**Keywords:** hernia prostheses, porosity, pore size, knitting structure, synthetic meshes

## Abstract

Inguinal hernia repairs are among the most frequent operations performed worldwide. This study aims to provide further understanding of structural characteristics of hernia prostheses, and better comprehensive evaluation. Weight, porosity, pore size and other physical characteristics were evaluated; warp knitting structures were thoroughly discussed. Two methods referring to ISO 7198:1998, *i.e.*, weight method and area method, were employed to calculate porosity. Porosity ranged from 37.3% to 69.7% measured by the area method, and 81.1% to 89.6% by the weight method. Devices with two-guide bar structures displayed both higher porosity (57.7%–69.7%) and effective porosity (30.8%–60.1%) than single-guide bar structure (37.3%–62.4% and 0%–5.9%, respectively). Filament diameter, stitch density and loop structure combined determined the thickness, weight and characteristics of pores. They must be well designed to avoid zero effective porosity regarding a single-bar structure. The area method was more effective in characterizing flat sheet meshes while the weight method was perhaps more accurate in describing stereoscopic void space for 3D structure devices. This article will give instructive clues for engineers to improve mesh structures, and better understanding of warp knitting meshes for surgeons.

## 1. Introduction

Hernias occur when parts of an organ protrude through the abdomen wall, causing defects in either the groin or abdomen. Worldwide, 20 million hernia surgeries are performed every year, and 80% of operations involve synthetic meshes [1]. Synthetic prostheses date back to 1948 when nylon was introduced, with the advent of polyester and polypropylene mesh in the 1950s [2,3]. Their use increased considerably after convincing tension-free hernioplasties [4]. Such surgical procedures are well accepted and considered necessary to relieve pain and prevent fatalities caused by strangulation of the intestines or other organs [5].

Prosthesis implantation, generally deemed to be superior to conventional methods, can eliminate strain, enable swift recovery and generate tissue ingrowth [6]. Many devices developed over the last few decades are now available commercially, resulting in a tremendous increase of prostheses types with various designs [7]. They include polypropylene (monofilament), polyester (multifilament) and polytetrafluoroethylene (membrane) polymers. Composite barrier patches are available as well: they are synthetic fabrics combined with a permanent or absorbable layer. This plethora of devices is a clear-cut indication that no consensus has been reached in identifying the most appropriate device for specific patients [8]. Under such circumstances, it can be understood why recognition of the most appropriate devices for these surgical procedures is still disputed. Hernia recurrence varies between 10% and 20% [9,10,11].

The US Food and Drug Administration (FDA) has recalled several devices over the last decade. Adverse events are nowadays analyzed carefully after individual reporting, more specifically through Manufacturer and User Facility Device Experience Database (MAUDE), set up by the FDA for surveillance in the US [12]. Any device implanted in the body requires continuous assessment. Adverse events must be reported and classified: device-related, patient-related and/or surgical technique-related.

Synthetic meshes are positioned to reinforce tissue defects [13], They should allow healthy tissue in-growth for incorporation into native tissues [14], retaining enough strength to withstand abdominal pressure [15,16] and enough elasticity under physiological pressure and corresponding anisotropy [17,18]. The mechanical properties of different devices have been explored intensively over the last few decades [19,20,21,22]. Mechanical mismatches with the abdominal wall, especially insufficient elasticity after implantation, still need to be addressed [17,19,23]. Great differences in anisotropy of various hernia prostheses are also under-appreciated [24], as well as the placement direction to avoid physiological mismatch [25]. The most influential factors impacting biological and mechanical properties warrant in-depth validation [26,27]. They cannot be addressed without a sound understanding of the structural characteristics of hernia mesh, because better mechanical performance must be achieved by good structure design. Undoubtedly, inappropriately-designed devices implanted in hernia repair will result in poor mechanical properties, contributing significantly to device-related complications.

Most hospitals can only stock a limited inventory owing to cost, space and shelf life constraints. Considering that there are more than 200 commercially available products and devices with various structures and designs, surgeons have the difficult task of selecting the most appropriate available for each hernia repair situation. Proper selection also requires an understanding of device characteristics. Regrettably, vocabulary mismatch between manufacturers, engineers and surgeons has not subsided over the years and still occurs [28]. Deeken *et al.* [19,22], Coda *et al.* [3] and Klinge and Klosterhalfen [29] have been pioneering the development of sound classifications and physico-mechanical characterizations in recent decades.

Pore size, thickness, weight, and mesh weave characteristics are the principal labels that characterize hernia prostheses that everyone can understand. They are also proposed in product descriptions by FDA guidelines in the USA for the preparation of premarket notification application for prostheses. Many clinical assessments, comparing heavyweight and lightweight devices or large and small pore size devices, can be found in the literature [30,31,32]. Lightweight and large pore meshes have been shown to have preferable clinical outcomes with less rigid scar tissue, shrinkage and chronic inflammatory reactions, manifesting softness on the abdominal wall and decreasing chronic pain in patients [33,34,35]. Although lightweight meshes undoubtedly compromise mechanical strength, large porous structures might enhance it *in vivo* with mature collagen type I attachment [36]. However, warp-knitting structures have been ignored and are not familiar to many researchers when they investigate physical or mechanical properties. Knitting structures have not only resulted in different porosities but also different mechanical properties, *i.e.*, strength, elastics, and anisotropy. It is important to comprehensively describe physical structures in terms that can be understood by both surgeons and engineers.

Various methods are employed to characterize the physical features of mesh. No standard, consensual test methods have been acclaimed for hernia prostheses. Consequently, available information about mesh porosity is somewhat cloudy and varies from 0% to 97% without clearly-described methods [37]. McDermott *et al.* [36] and Pourdeyhimi [38,39] proposed porosity measurements based on area ratio to express void volume. Mühl *et al.* [37] developed a new approach to analyzing pore characteristics, taking 1 mm as effective pore size into consideration. We hereby propose a comprehensive analysis of physical structures, and employ a simple objective method to measure the porosity of medical textile prostheses designed for hernia repair, with warp knitting structure expounded as well. Mechanical test is not within the scope of this study, but mechanical investigation is planned in our future hernia mesh research protocol. The abdominal wall shows anisotropy behavior with greater strength in transversal direction than longitudinal direction [18,40,41] and more elasticity in longitudinal direction than in the transversal direction [17,42]. Prostheses should be placed in the proper direction to comply with the anisotropy of abdominal wall under physiological pressure. We anticipate further understanding of the structural characteristics of hernia prostheses with warp knitting structure in lay but precise terms for surgeons and medical researchers. More attention should be paid to structure so that corresponding mechanical investigations could give advice on improved designs possessing better biocompatibility, stability and compliance. 

## 2. Results

### 2.1. Textile Structure Characteristics

All devices examined here were warp-knitted fabrics. Their structures were formed by interlacing filament loops. They were different from woven fabrics, which were formed by the lengthwise yarns and widthwise yarns crossing each other. Warp knitting does not limit the use of only 1 guide bar in structure design: multi-guide bars can create more sophisticated fabric patterns and produce symmetric loop structures as well as larger pores. Table 1 reports the filament diameter, linear density and stitch density of fabrics, and Table 2 lists the results of loop structures and knitting diagrams along with light microscopic observations. Table 1, textile structures and physical characteristics of fabrics.

**Table 1:** Textile structures and physical characteristics of fabrics.

Device No.	Filament Diameter (mm)	Filament Linear Density (Tex)	Stitch Density(cm)		Thickness (mm)	Weight (g/m^2^)	Porosity (%) Area Method	Porosity (%) Weight Method
			Wales	Courses				
1	0.165 ± 0.005	19.25	5.5 ± 0.0	22.0 ± 0.3	0.71 ± 0.01	97.0 ± 0.0	41.6 ± 1.1	84.7 ± 0.0
2	0.114 ± 0.004	9.19	7.1 ± 0.1	14.5 ± 0.2	0.36 ± 0.01	–	62.4 ± 1.3	–
3	0.162 ± 0.002	18.55	6.0 ± 0.0	21.9 ± 0.2	0.66 ± 0.01	99.8 ± 2.0	40.1 ± 1.0	83.2 ± 0.3
4	0.121 ± 0.013	10.35	5.0 ± 0.0	13.2 ± 0.0	0.43 ± 0.03	40.1 ± 0.1	64.8 ± 0.8	89.6 ± 0.0
5	0.102 ± 0.001	7.36	9.0 ± 0.0	15.9 ± 0.1	0.35 ± 0.01	35.9 ± 0.0	57.7 ± 0.6	88.7 ± 0.0
6-1	0.157 ± 0.006	17.52	7.5 ± 0.1	17.4 ± 0.1	0.60 ± 0.01	90.3 ± 0.1	44.8 ± 1.1	83.3 ± 0.0
6-2	0.163 ± 0.005	18.73	5.5 ± 0.0	22.0 ± 0.2	0.68 ± 0.01	90.9 ± 0.1	41.5 ± 0.5	85.2 ± 0.0
7	0.156 ± 0.002	17.20	7.0 ± 0.1	18.0 ± 0.3	0.57 ± 0.02	97.2 ± 4.6	37.3 ± 1.1	81.1 ± 1.0
8	0.092 ± 0.005	5.98	12.0 ± 0.0	20.0 ± 0.1	0.29 ± 0.00	35.0 *	60.4 ± 0.7	–
9	0.065 ± 0.001	2.95	11.2 ± 0.1	19.2 ± 0.1	0.22 ± 0.00	16.0 *	69.7 ± 0.6	–

* Weight from device specification.

#### 2.1.1. Filament Diameter 

All devices were manufactured with monofilament. Filament diameters ranged from 0.065 mm to 0.165 mm. TiMESH extralight, which is a lightweight device, displayed the smallest value with a diameter of 0.065 mm, and Bard Mesh Large Pre-shaped with Keyhole exhibited the largest value with a diameter of 0.165 mm, which was 2.5 times larger than the thinnest diameter. 

#### 2.1.2. Linear Density

Linear density of the filaments was calculated for seven devices considering that TiMESH light and TiMESH extralight were not pure polypropylene because of titanium oxide coating on the filament surface. Values ranged from 7.36 to 19.27 tex, which reflects the mass amount per unit length of filament. The unit “tex” is defined as the mass in grams per 1,000 meters.

#### 2.1.3. Stitch Density

The wale density of all devices ranged from 5.0 to 12 wales/cm, and course density ranged from 13.2 to 22 courses/cm. Consequently, the number of loops per cm^2^ were evidently distinct from each other.

#### 2.1.4. Loop Structures and Knitting-Lapping Diagrams

The structures appearing in Table 2 illustrate a variety of constructions, from compacted to wide open, resulting in different pore shapes, pore size and pore distributions.

Six single, fully-threaded guide bar structures in these devices were studied—No. 1: Bard Mesh Large Pre-shaped with Keyhole; No. 2: Bard Polysoft Hernia Patch; No. 3: Bard Mesh Pre-shaped; No. 6-1: Autosuture Surgipro Mesh; No. 6-2: Autosuture Surgipro Mesh; No. 7: Holypro—along with four two-guide bar structure fabrics (No. 4: Bard Soft Mesh Pre-shaped; No. 5: Parietene PPL0611X3; No. 8: TiMESH light; No. 9: TiMESH extralight).

**Figure 1:**
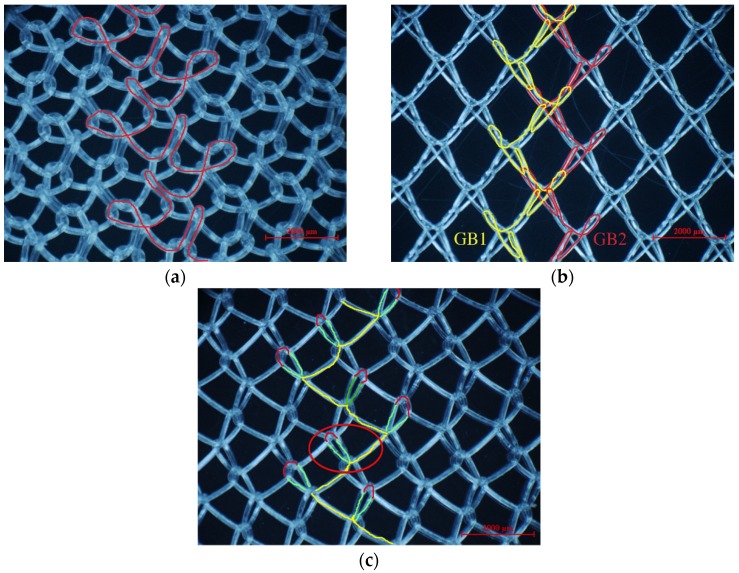
Illustrations for warp-knitting structure. (**a**) Device No. 6-1; (**b**) Device No. 9; (**c**) Device No. 2.

**Figure 2:**
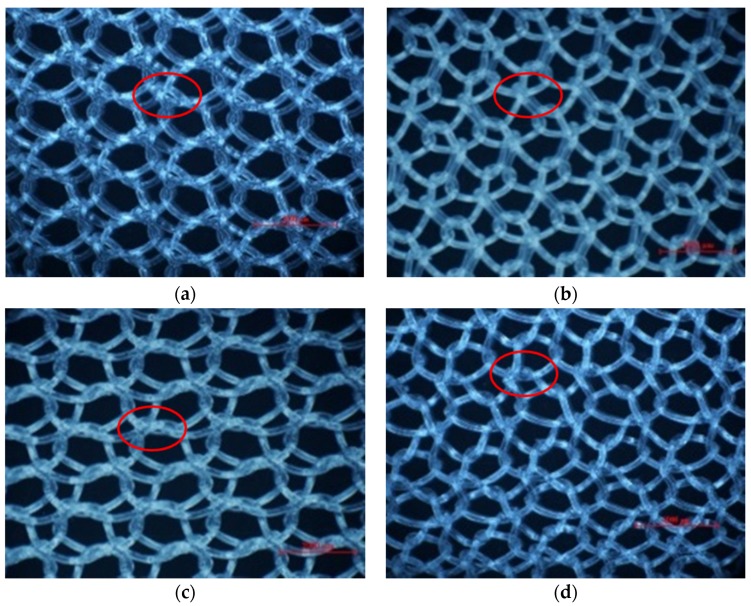
Differences in open loops of the same lapping structures shown in red circles. (**a**) Device No. 3; (**b**) Device No. 6-1; (**c**) Device No. 6-2; (**d**) Device No. 7.

In warp knitting, each filament is threaded through the hole of a guide, with a row of guides cast together and attached to a guide bar. It is the guide bar swing and shogging mechanism that forms the overlap and underlap paths of a filament end around the needles. All guides in a guide bar produce an identical lapping movement, so single-guide bar structures possess only one regular pattern of each filament, as shown by the red line in Figure 1a. Two guide bars can produce two kinds of lapping movement, so two-guide bar structures possess two kinds of regular filaments pattern, as illustrated by the red and yellow line in Figure 1b. Guide bar lapping movement are composed of overlapping and underlapping movement. A complete loop comprises three parts: an overlap formed by overlapping movement (red lines in Figure 1c), an underlap formed by underlapping movement (yellow lines in Figure 1c), pillars formed by swing movement (green line in Figure 1c). Progressive lapping can be closed-lap, as depicted by the red circle in Figure 1c and can also be open-lap, as shown by the red circle in Figure 2 as well.

No. 1, No. 3, No. 6-1, No. 6-2 and No. 7 exhibited the same structure pattern design with an open-lap three-needle atlas structure, which is a lapping movement where the guide bar makes an underlapping movement across three needles with two courses in the same direction, followed by an identical lapping movement in the opposite direction. However, open-lap morphology is obviously different for device No. 3, No. 6-1, No. 6-2 and No. 7, as seen in Figure 2. Device No. 2 displayed a closed-lap three-needle atlas lapping structure. It was the different loop morphologies that give different pore characteristics.

Considering four devices with two-guide bar structures, devices No. 8 and No. 9 possess the same knitting-lapping structure, devices No. 4 and No. 5 possess two other knitting-lapping structures. These four devices exhibit a symmetrical net structure. Devices No. 8 and No. 9, which have diamond-shaped nets, were formed by two guide bars moved in opposite direction with movement path of open-lap three-needle atlas structure and a rule of 1 full, 1 empty threaded through a line of needles. Device No. 4, which was a hexagonal-shaped net with inlay filament effect, was achieved by closed laps and a variation of underlaps. Device No. 5 presented the most sophisticated structure, the pore shapes are comprised of quadrangle net-shapes and triangle net-shapes.

**Table 2:** Loop structures and knitting-lapping diagrams.

Device No.	Structure	Light Microscopy (20×)	Knitting-Lapping Diagram	Chain Notation
1	single-guide bar; open-lap three-needle atlas	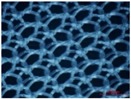	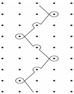	1-0/1-2/2-3/2-1//
2	single-guide bar; closed-lap three-needle atlas	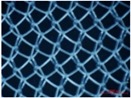	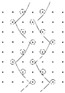	1-0/2-1/2-3/1-2//
3	single-guide bar; open-lap three-needle atlas	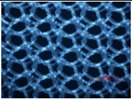	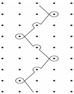	1-0/1-2/2-3/2-1//
4	two-guide bars; modified tricot	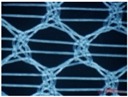	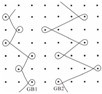	GB1:2-3/2-1/2-3/1-0/1-2/1-0// GB2:2-1/4-3/2-1/3-4/1-0/2-1//
5	two-guide bars; combined atlas and tricot lapping	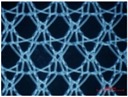	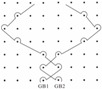	GB1:3-4/5-4/4-3/2-1/0-1/1-2// GB2:2-1/0-1/1-2/3-4/5-4/4-3//
6-1	single-guide bar; open-lap three-needle atlas	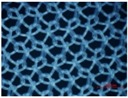	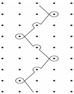	1-0/1-2/2-3/2-1//
6-2	single-guide bar; open-lap three-needle atlas	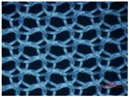	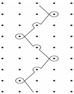	1-0/1-2/2-3/2-1//
7	single guide bar; open-lap three-needle atlas	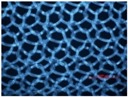	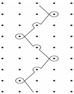	1-0/1-2/2-3/2-1//
8	two-guide bars; open-lap three-needle atlas	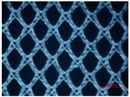	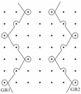	GB1:1-0/1-2/2-3/2-1// GB2:2-3/2-1/1-0/1-2//
9	two-guide bars; open-lap three-needle atlas	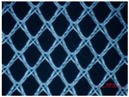	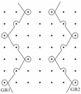	GB1:1-0/1-2/2-3/2-1// GB2:2-3/2-1/1-0/1-2//

### 2.2. General Physical Properties

The different general physical properties listed in Table 1 resulted from different textile structure characteristics.

#### 2.2.1. Thickness 

Thickness was calculated as an average fabric value, because interlacing nodes of the loops were undoubtedly higher than underlap loops. It ranged from 0.22 mm to 0.71 mm for different knitting structures. The thickest fabric was device No. 1 while the thinnest was device No. 9.

#### 2.2.2. Density

Unit mass varied from 35.9 g/m^2^ to 99.8 g/m^2^ for porous textile structures except for device No. 2, No. 8 and No. 9, which could not be calculated from the method described in Section 4.2.3 because of the self-expanding ring in the periphery of No. 2, and titanium coating on the filament of No. 8 and No. 9. The weights of device No. 8 and No. 9 are given in their specifications in Table 1. The lightest was device No. 9, and the heaviest was device No. 3.

#### 2.2.3. Porosity

The porosity of devices No. 2, No. 8 and No. 9 could not be calculated by the weight method mentioned in Section 2.1. Porosity values ranged from 37.3% to 69.7% by the area method, and from 81.1% to 89.6% by the weight method. It was obvious that porosity measured by the weight method for each device was quite higher than that measured by the area method. Device No. 9 exhibited maximum porosity of 69.7% by the area method. Device No. 7 displayed minimum porosity of 37.3% by the area method as well as minimum porosity of 81.1% by the weight method.

**Figure 3:**
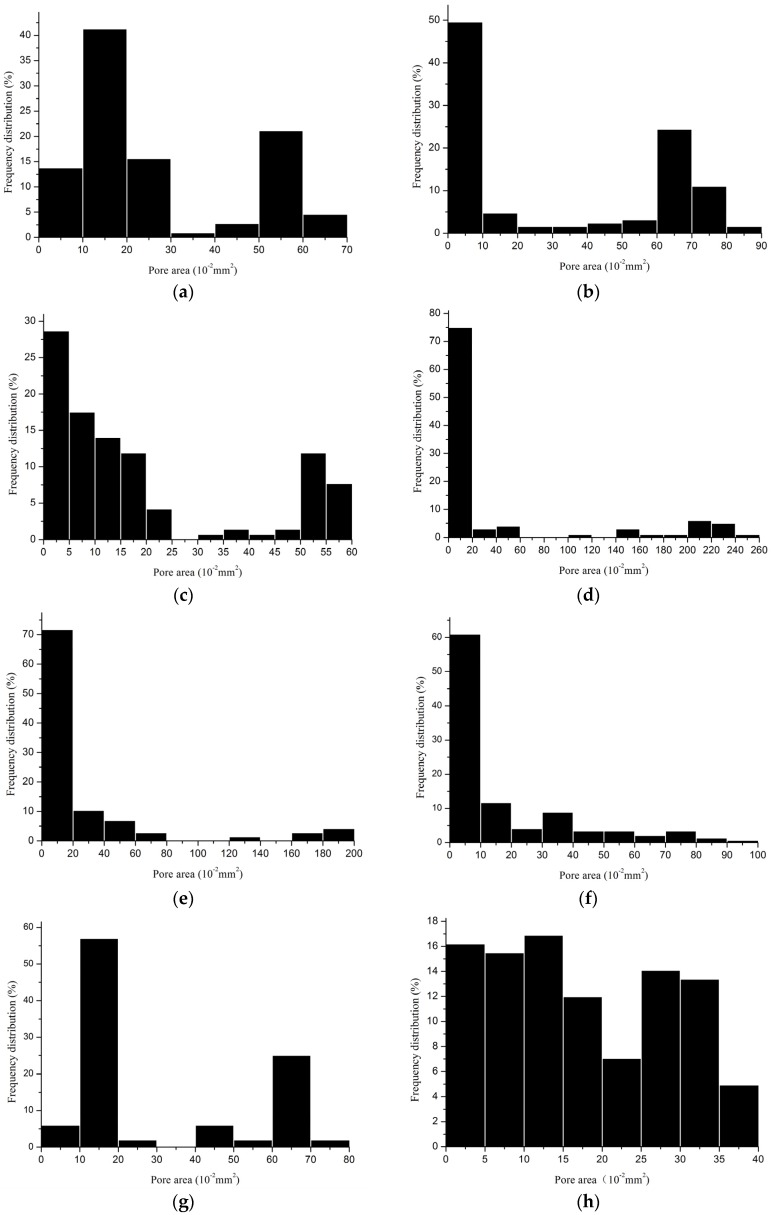

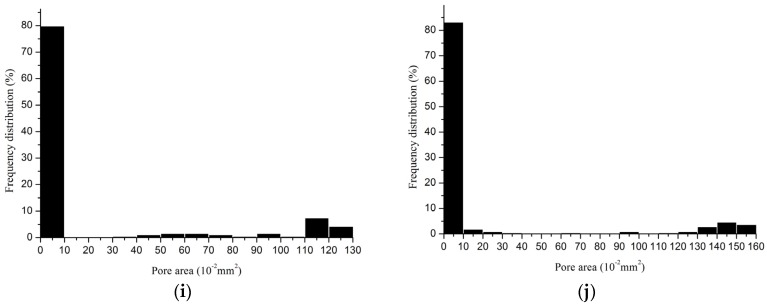
Distribution of pores in all devices. (**a**) Device No. 1; (**b**) Device No. 2; (**c**) Device No. 3; (**d**) Device No. 4; (**e**) Device No. 5; (**f**) Device No. 6-1; (**g**) Device No. 6-2; (**h**) Device No. 7; (**i**) Device No. 8; (**j**) Device No. 9.

The pore size distributions of all devices were analyzed according to the processing method in Section 4.2.3 and the results reported in Figure 3. Distribution graphs represent results selected randomly from five repeated examinations. They show the proportion of pores within a certain pore area, e.g., in device No. 1, the maximum pore area was 0.65 mm^2^, and pore areas ranged from 0.1 to 0.2 mm^2^ in a proportion of 41.3%. Maximum and minimum pore areas as well as porosity corresponding to the above-mentioned examinations are listed in Table 3 and Table 4. Maximum pore areas ranged from 0.39 mm^2^ to 0.98 mm^2^ in single-bar structure meshes, compared to 1.58–2.40 mm^2^ in two-guide bar structure meshes, demonstrating apparent differences in pore sizes between single-guide bar and two-guide bar structures. Currently, minimum pore area was more a filament interstice than a genuine pore; therefore, it was the maximum pore area that gave information on the largest void content in a device. The area (0.785 mm^2^) of a circle with a diameter of 1 mm was identified as an effective distance between filaments to prevent the bridging effect. The porosity of pore areas larger than or equal to 0.785 mm^2^ from the representative results are calculated in Table 3 and Table 4 and named porosity I, ranging from 0% to 60.1%. Device No. 9 displayed the largest value, and four single-bar structure devices (No. 1, No. 3, No. 6-2 and No. 7) showed zero value. In addition, two other single-bar structure devices, No. 6-1 and No. 2, presented extraordinarily low values (5.1% and 5.9%, respectively) compared to the other four two-guide bar structure devices.

**Table 3:** Pore size and porosity (area method) of single-bar structure devices.

Device	Minimum Pore Size (mm^2^)	Maximum Pore Size (mm^2^)	Porosity (%)	Porosity I * (%)
1	0.0007	0.6500	42.6	0
2	0.0018	0.8700	61.7	5.9
3	0.0040	0.5899	40.5	0
6-1	0.0065	0.9827	44.4	5.1
6-2	0.0096	0.7300	42.1	0
7	0.0012	0.3916	37.3	0

* Porosity of pores whose area is larger than 0.785 mm^2^.

**Table 4:** Pore size and porosity (area method) of two-guide bar structure devices.

Device	Minimum Pore Size (mm^2^)	Maximum Pore Size (mm^2^)	Porosity (%)	Porosity I * (%)
4	0.0042	2.400	64.2	54.7
5	0.0044	1.900	58.2	30.8
8	0.0052	1.2517	60.1	46.2
9	0.0013	1.5776	69.2	60.1

* Porosity of pores whose area is larger than 0.785 mm^2^.

### 2.3. Relationship between Different Properties of the Devices

**Figure 4:**
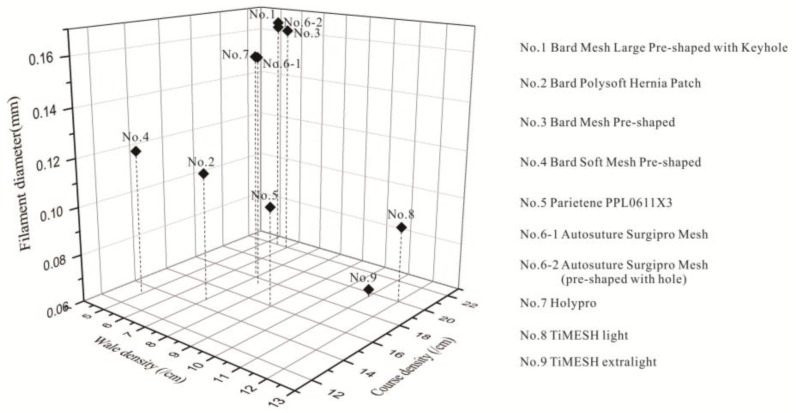
Relationship between filament diameter and stitch density of hernia prostheses.

Figure 4 illustrates the stitch density and filament diameter of each device, considering same loop structures, such as device No. 1, No. 3, No. 6-1, No. 6-2 and No. 7. Devices with lower filament diameters had larger wale density, except for device No. 6-1 and No. 6-2, which had an apparently distinct open-lap morphology on microscopy observations (Figure 2). Device No. 8 and No. 9 not follow the law of lower filament diameter possessing larger wale density, notwithstanding their same knitting-lapping structures. They were the result of different machine gauge and warp run-in during processing.

Considering the relationship between thickness and weight per square meter, devices No. 5, No. 4, and No. 7 showed significant positive correlations: thicker mesh weighed more, as with devices No. 6-1 and No. 6-2. However, devices No. 1 and No. 3 were different and weighed comparably with minor variances in thickness. Device No. 8 (35 g/m^2^), with larger filament diameter and stitch density, weighed more than double device No. 9 (16 g/m^2^), as given by their specifications. It can be seen from Figure 4 that the thickness is not determined only by weight. Thickness and weight are the consequence of filament diameter and stitch density. All textile structure properties combined determined the physical feature of warp knitting mesh.

**Figure 5:**
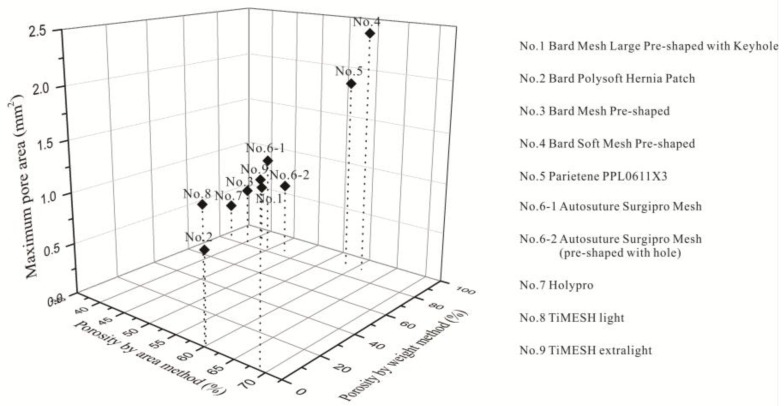
Maximum pore areas and porosities by the two measurement methods: area *vs.* weight.

Figure 5 is a 3D scatter graph of maximum pore sizes and porosities measured by two methods, illustrating clear-cut evidence of differences in various structures. Porosities of devices No. 1, No. 3, No. 4, No. 5, and No. 7, measured by the weight and area methods, presented a similar trend, even though apparent differences existed in porosity values calculated by these methods. Trend of device No. 6-1 and No. 6-2 were converse with the 2 methods. Although the thickness and weight of device No. 6-2 was slightly larger than device No. 6-1, stitch density per cm^2^ was smaller than device No. 6-2. Consequently, material ratio in the same size of stereoscopic void space of device No. 6-2 was smaller than No. 6-1, resulting in larger porosity of device No. 6-2 by the weight method. It was observed that the ratio of wale density to course density of device No. 6-1 was almost twice that of device No. 6-2. Therefore, slanting tendency of loops of device No. 6-1 and No. 6-2 were different. This factor, combined with a larger filament diameter, will have an impact on their surface ratio of material. The larger porosity of No. 6-1 compared to No. 6-2 by area method is merely the surface void.

Device No. 4 displayed not only the largest pore size (2.40 mm^2^) of all these devices, but also considerably higher porosity (64.2%). Device No. 7 exhibited the smallest maximum pore size (0.39 mm^2^) and lowest porosity (37.3%) by the area method, indicating that devices with the highest porosities did not necessarily possess the largest pore sizes. The porosity (61.7%) of device No. 2 was apparently greater than that of devices No. 1, No. 3, No. 6-1, No. 6-2 and No. 7 (37.3%–44.4%), indicating that closed-lap three-needle atlas structure exhibit larger porosity than open-lap three-needle atlas structures. However, the larger porosity of device No. 2 benefits from its small stitch density as well. It was evident that the great decreased filament diameter and a slightly smaller stitch density can increase both porosity and maximum pore size from devices No. 8 and No. 9.

## 3. Discussion

The purpose of this research was to provide a better understanding of mesh structural characteristics to both surgeons and engineers with comprehensive evaluation of the physical characteristics of meshes. The results reported here do not open clear-cut avenues and point out differences in comparisons of various device issues. Multiple questions can still be raised about the physical characteristics of hernia prostheses, lapping structures and pore features as well as effectiveness of the method selected to measure the porosity itself. Let us address these issues serially.

### 3.1. Physical Characteristics of Hernia Meshes

Most man-made polymers in fabric prostheses were rapidly employed as implants after they were synthesized as alternatives to natural fibers: nylon in 1935 by Carothers, rapidly followed by dacron (polyester or polyethylene terephthalate) in 1941 by Whinfield and Dickson, and polypropylene in 1954 by Natta and Zeigler [43]. Clinical applications followed shortly thereafter for nylon in 1948 [44] and for dacron in 1956 [45] and polypropylene in 1959 [46]. Manufacturers produce hernia prostheses with different features by selecting monofilament diameter and knitting structure. Diameter determines monofilament strength, which, in turn, influences fabric strength. However, monofilament diameter must be optimized because of bending rigidity increased by growing diameter. Handling characteristics rapidly becomes inappropriate with larger monofilament diameter, as does adaptability to implantation site. Polypropylene, reported here, is the filament used most often in hernia prostheses and is commonly considered to be inert. However, recent studies had confirmed the polypropylene filament degradation with evident surface cracking after one year of implantation, leading to brittleness and loss of flexibility [47,48]. The issue of polypropylene biostability has been overlooked, despite abundant literature reports, and much attention should be paid to materials. The *in vivo* biostability of polyvinylidene fluoride is more acceptable than that of polypropylene [49,50].

Filament diameters, for knitting or weaving hernia prostheses, therefore play an important role in the physical structure of meshes. Normally, stitch density is influenced by filament diameter when knitting structure design and manufacture parameters are same. Stitch density is larger with smaller filament diameter. In addition, if loop structure and stitch density are identical, porosity will be larger as well with thinner filaments. However, the relationship between filament diameter and stitch density of all devices could not be observed because of other influential factors mentioned above. From the fabrication point of view, the wale density of warp knitting fabric is closely related to machine gauge and knitting structure, whereas course density is relevant to warp run-in. In addition, the fabric finishing process will contribute to fabric structure stability as well as fabric handling. Stitch densities are influenced by tension in heat-setting finishing process as well and, thus, have an impact on mesh weights.

The thickness and weight of knitted meshes are impacted not only by linear density but also by fabric stitch density and loop structure. Mesh weight is an important and influential factor responsible for biocompatibility due to material amount used in surgery, which sway the extent of interactions between prosthesis and host. Devices No. 8 and No. 9 demonstrated that lightweight meshes can be achieved with thinner filament, smaller machine gauge and warp run-in during processing, thus contributing to reduction of foreign material reactions [51]. Earle and Mark [52] classified hernia prostheses into ultra-light (<35 g/m^2^), light (35–50 g/m^2^), medium (51–90 g/m^2^) and heavy (<90 g/m^2^) weight mesh, while Coda *et al.* [3] proposed a new classification based on weight in 2012, and divided all studied devices into ultralight (<35 g/m^2^), light (≥35 g/m^2^, <70 g/m^2^), standard (≥70 g/m^2^, <140 g/m^2^) and heavy weight (≥140 g/m^2^) meshes. Interestingly, all two-guide bar structure devices were lightweight meshes. All single-bar structure devices except device No. 2 without calculated value were heavy weight meshes, according to the former classification, but were standard weight meshes, according to the new classification.

Considering four devices with two-guide bar structures, it was observed that device No. 4 had considerably larger porosity with the largest pore area despite possessing the largest filament diameter and thickness. Device No. 8 has larger filament diameter, thickness, stitch density than device No. 9, which led to smaller maximum pore area and porosity. Device No. 5 displayed the lowest porosity in these two-guide bar structure devices despite weighing less than device No. 4. It can be concluded from these observations that two-guide bar structure mesh has larger pore size than single-bar structure mesh. Even though device No. 2 had larger porosity than other single-bar structure devices, it would be ambiguous to conclude that closed-lap structures are better than open-lap structures for three-needle atlases when considering pore size, because the stitch density of device No. 2 was much smaller than that of other open-lap three-needle structures. Besides, open-lap three-needle atlas structures, e.g., device No. 6-1 presented larger pores by adjusting other parameters in structure design.

Filament diameter, stitch density and loop structure not only determine the weight and porosity, but also will have an impact on mechanical property and anisotropy. Although this article contains no mechanical test, theoretically, curved loops begin to be straight and knots of loops begin to move but are not stretched in the first stage of tensile strength testing for warp knitting textiles. Consequently, loop numbers in longitudinal direction will influence longitudinal elasticity and loop numbers in horizontal directions will impact horizontal elasticity. Mechanical behavior will also be different for prostheses with different loop patterns, because meshes with two-guide bar structures sometimes have symmetrical loop structures, the differences between longitudinal and horizontal directions being smaller than single-guide bar structures. Understanding of structures is beneficial for surgeons and researchers, because anisotropy should comply with the abdominal wall to avoid mechanical mismatch. We hope the elaboration of structures will help researchers and surgeons not familiar with warp knitting to know more about mesh structure, thus paying more attention to structure when investigating mechanical properties and placing mesh in which direction along the longitudinal direction of abdominal wall in surgery.

### 3.2. Pore Characteristics

Fabrics for abdominal wall reinforcement or repair should behave like scaffolds, permitting cells to adhere and tissue to grow, thereby enhancing their strength. Pore size and distribution are key factors not only for handling prostheses during operations but also for promoting adequate tissue incorporation and healing tissue encapsulation. They have been perceived as significant physical properties of hernia mesh, exerting tremendous influence on mesh biocompatibility. Therefore, the issue needs to be settled with rational design.

In distribution graphs, area range was the smallest distributed with the largest proportion of all pores, except for device No. 1, No. 6-2 and No. 7, but the area range mentioned above for these three devices was also quite small. Obviously, two-guide bar structures displayed a much larger proportion of pores distributed in a small area range than single-bar structure devices. Apparently, such large proportions cannot be recommended, but the correspondingly smallest areas were undoubtedly the interstices between interlaced filaments. Another noteworthy observation was the proportion of pores in a larger area range, because pores with larger area range in device were the exact macroporous pores.

It has been shown that single-bar structures display a larger proportion of pores distributed in large areas than two-guide bar structures. However, it cannot be considered as being better for pore characteristics only according to this distribution. Another important consideration is the percentage of pore area larger than or equal to 0.785 mm^2^, as mentioned above. In distribution graphs, it is evident that two-guide bar structures exhibit a higher percentage of pores larger than 0.785 mm^2^, and many single-guide bar structures even display zero values. Although the smallest distance leads to fibrous bridging, distribution of very large pores in fabrics could contribute to more elasticity. Furthermore, it was distributed more evenly in different area ranges for device No. 7, so that the pores were neither too small nor too big.

Many devices on the market are described as small pore, medium pore, or large pore meshes without evident boundaries to classification. According to the new classification proposed by Klinge *et al.* [29], four devices were large pore meshes (porosity > 60%), including three two-guide bar structure devices (No. 4, No. 8 and No. 9) and a single-bar structure device (No. 2). Six devices were small pore meshes (<60%), including one two-guide bar structure device (No. 5) and five single-bar structure devices (No. 1, No. 3, No. 6-1, No. 6-2 and No. 7). However, large pore meshes can be ranked by effective porosity as well. Although the porosity I results differ from porosity measured by the iterative method as proposed by Mühl *et al.* [37], porosities taking 1-mm distance into account were much lower than porosities of all interstices in fabrics, even going as low as zero. Based on these considerations, all two-guide bar structure devices displayed higher porosity (30.8%–60.1%) than single-guide bar structure devices (0%–5.9%). All two-guide bar structures are large pore size meshes, and only two devices (No. 2 and No. 6-1) with single-guide bar structures can be considered as large pore size meshes as well. 

### 3.3. Why Can the Same Lapping Structure Lead to Different Pore Characteristics?

Four devices had the same knitting-lapping structure, as shown in Figure 1, but displayed a different morphology of open loops, as indicated by red circles. The open loop of No. 6-2 was smaller than that of No. 6-1 and No. 7, resulting from different needles (latch needle or compound needle) on the machine. Differences between No. 3 and No. 6-2 and between No. 6-1 and No. 7 were closely related to stitch density and warp yarn tension during fabrication, leading to different slanting tendency of the open loop. Extension during heat-setting of fabrics greatly contributes to open loop morphology as well. It was the different loop morphologies in the same loop structure that result in different porosity and pore size distribution.

Interestingly, No. 6-1 was the only device among open-lap three needle atlas, single-guide bar structure devices that displayed a porosity I with more than zero. Its pore morphology demonstrated that long and tight open loops for open-lap atlas structures have the advantage of better pore characteristics compared to other open-lap atlas structures. Furthermore, device No. 2 was another mentioned single-bar structure: it exhibited not only much larger porosity than other single-bar structure devices, but also the largest porosity I, providing another strategy to design better pore characteristics for single-guide bar structures, *i.e.*, closed-lap three-needle atlas structures with smaller stitch density. However, other devices with closed-lap three-needle atlas structures need to be investigated to confirm this conclusion.

Conclusively, when manufacturing a warp knitting mesh, the stitch density, filament diameter and morphology of the open-lap loops while employ single-guide bar structures should be designed appropriately to avoid the device possessing an effective porosity I of zero value.

### 3.4. Which Method Could Be Most Effective in Characterizing the Pore Size of Hernia Prostheses?

Although porosity has long been discussed by researchers and deemed to be the most influential property for biocompatibility and abdominal wall compliance, there is no standard classification and calculation of porosity. Porosity measurements by the weight and area methods reported here were adapted from those for cardiovascular implants, namely, tubular vascular prostheses. Both methods are acceptable for measuring the porosity of knitting meshes. The area method describes more characteristics of pores but is limited to devices with 3D structures, because it does not take spatial structure into account, presenting a void ratio of material-tissue surface. In contrast, the weight method may be more accurate when comparing 3D structure devices, because it gives the void ratio of stereoscopic void space used in the body. However, it does not provide any clue about void distribution and pore size. The results and analyses of these two methods indicated that both could be employed to make porosity comparisons between different flat prostheses when the meshes are not close in thickness or weight. For devices with nearly equivalent thickness and weight as well as same knitting-lapping structure, different wale density and stitch density make a different slanting tendency of loops, resulting in different height and surface area in the knotting part of loops. Consequently, the devices with more stereoscopic void space do not absolutely display a larger surface void space. As pore size plays a significant role in the biocompatibility and the elasticity of mesh-tissue integration after implantation, the area method has many advantages in depicting pore distribution, maximum pore size and porosity when taking 1-mm distance into consideration. 

Therefore, the area method is indisputably the most appropriate for evaluating the pore characteristics of flat sheet meshes. Finally, the possibility of analyzing structures in 3D porous biomaterials with Micro-CT scan machines deserves attention in future research [53,54,55].

## 4. Materials and Methods

### 4.1. Selection of Devices

Table 5 lists nine devices that are representative of the most frequently-implanted prostheses for hernioplasties. They are manufactured in USA by Bard (Warwick, RI, USA) and US Surgical (Norwalk, CT, USA), in France by Sofradim (Trevoux, France), in Germany by GfE Medizintechnik GmbH (Nuremberg, Germany) and in China by Holycon Medical Instrument Co., Ltd. (Nantong, China). Seven devices are made of pure polypropylene monofilaments, and TiMESH Gfe patches are titanized. Polypropylene titanization occurs at low temperatures by a special plasma-coating process known as plasma-activated chemical vapor deposition [56,57].

**Table 5:** Hernia prostheses selected.

Device	Company	Product Name	Gross Observation	Type of Materials	Filament Type
1	Bard	Bard Mesh Large Pre-shaped with Keyhole	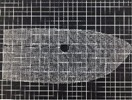	Polypropylene	Monofilament
2	Bard	Bard Polysoft Hernia Patch	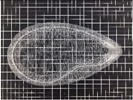	Polypropylene	Monofilament
3	Bard	Bard Mesh Pre-shaped	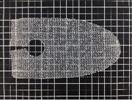	Polypropylene	Monofilament
4	Bard	Bard Soft Mesh Pre-shaped	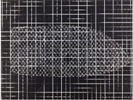	Polypropylene	Monofilament
5	Sofradim Production	Parietene PPL0611X3	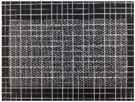	Polypropylene	Monofilament
6-1	Covidien/Medtronic	Autosuture Surgipro Mesh	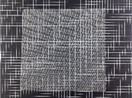	Polypropylene	Monofilament
6-2	Covidien/Medtronic	Autosuture Surgipro Mesh	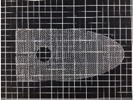	Polypropylene	Monofilament
7	Holycon Medical Instrument Co., Ltd.	Holypro	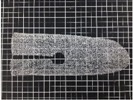	Polypropylene	Monofilament
8	GfE Medizintechnik	TiMESH light	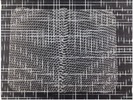	Titanized polypropylene	Monofilament
9	GfE Medizintechnik	TiMESH extralight	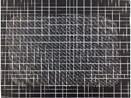	Titanized polypropylene	Monofilament

### 4.2. Methods

#### 4.2.1. Surface Morphology

Gross observations: Each device was examined and photographed with a digital camera (Sony HDR-XR160E, 4.2 Mega Pixels, Sony Corporation, Beijing, China).

Light microscopy: Specimens were observed at 20× magnification with a light compound SMZ745T microscope (Nikon Imaging (China) Sales Co. Ltd., Shanghai, China) fitted with a CCD Digital Sight DS-Fil camera. Images were processed with Adobe Photoshop CS.

#### 4.2.2. Textile Structure Characteristics

They included filament diameter, linear density, stitch density and the knitting structures of each device. Each test was repeated five times.

Filament diameter: Ten filaments were randomly selected from 10 different locations. They were photographed with a CH-2 optical compound microscope (Nikon Imaging (China) Sales Co., Ltd.) fitted with a CCD camera at 100× magnification. Each filament diameter was measured by MB-Ruler software according to the ratio between its length on the computer screen and its genuine scale bar length.

Linear density: This was calculated according to filament diameter measured by the above-mentioned method and the formula $d=0.03568\times \sqrt{N_{t}/\delta_{y}}$, in which *Nt* is linear density (tex) and δ*y* is mass density of polypropylene (0.9 g/cm^3^).

**Figure 6:**
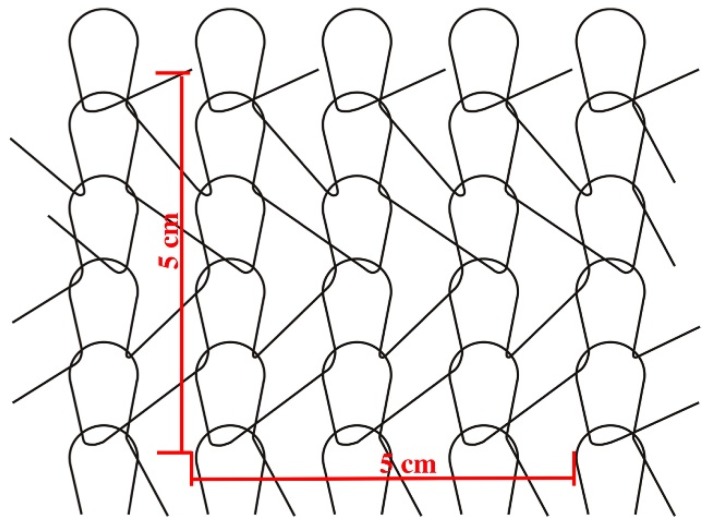
Diagram of stitch density measurement.

Stitch density: The number of stitches in the fabric was determined by counting the number of wales along 5 cm of fabric width, and along 5 cm of fabric length, as shown in Figure 6. Counted values were divided by five to get wale density (wales/cm) and course density (courses/cm).

Knitting structure: The knitting-lapping diagram and chain notation of each specimen were drawn by CorelDRAW Graphics Suite X7, according to loop-forming shape and interlaced structures. The lapping diagram represents loop formation on the knitting machine, in which points in horizontal rows denote needles in plain view, and loops along the vertical direction in the lapping diagram exemplify loop formations one course after another. This cyclic movement gives the pattern structures of warp-knitting fabrics, and loops of warp-knitting fabric are formed by guide bar movement in spaces between needles. The chain link reflects the shogging movement of each guide bar in producing particular fabric patterns.

#### 4.2.3. Physical Characteristics

They include thickness, density, and porosity by the weight and surface area methods.

Thickness: The thickness (mm) of each mesh was measured by a digital fabric thickness instrument, YG141N (Shanghai Liuling Instrument Factory, Shanghai, China), according to the Chinese standard textile test method adapted from ISO 5084:1996.

Density: The weight mass of five coupons were measured by FA2004 electronic analytical balance (Shanghai Wanning Precise Instrument Co., Ltd., Shanghai, China) to 0.1 mg resolution. Density was determined by mass per unit area (g/cm^2^) of fabric. The area of devices was measured by the ratio of pixel value of the devices *vs.* the selected rectangle area. The genuine area of the selected rectangle on computer screen can be measured by ruler, so the genuine area of devices can be calculated from the ratio value.

Porosity: Two methods, referring to ISO 7198:1998, were employed in our laboratory protocol to measure the porosity of hernia meshes. They represent measurement understanding with the goal of highlighting an effective way comparing the various features of devices.

Weight method: The porosity (P) of fabrics, *i.e.*, void space volume as a percentage of total fabric volume, was calculated as: *P* = 100 × (1 – *M*/*t*·ρ), where *M* was mass per unit area (g/cm^2^), *t* was fabric thickness (cm), and ρ was polypropylene density (0.9 g/cm^3^) [58].

Area method: Optical micrographs served as planimetric determinations of porosity. It was calculated as the ratio of total area of voids *vs.* the total area of voids and materials [58]. The porosity (*P*) of fabrics was determined here by pixel values of the whole picture photographed with 20× magnification, and total pixel values of pore areas were selected by Magic Wand Tool in Photoshop software. They were calculated as the ratio of total pixel value of pore areas to the pixel values of the whole picture. Furthermore, the genuine area of the portion photographed was calculated by the ratio of picture length and width on the computer screen to scale bar length on the image. Finally, the genuine area of each pore was calculated by the ratio of the pixel value of each pore to the pixel values of the whole image and the genuine area of this whole image.

**Figure 7:**
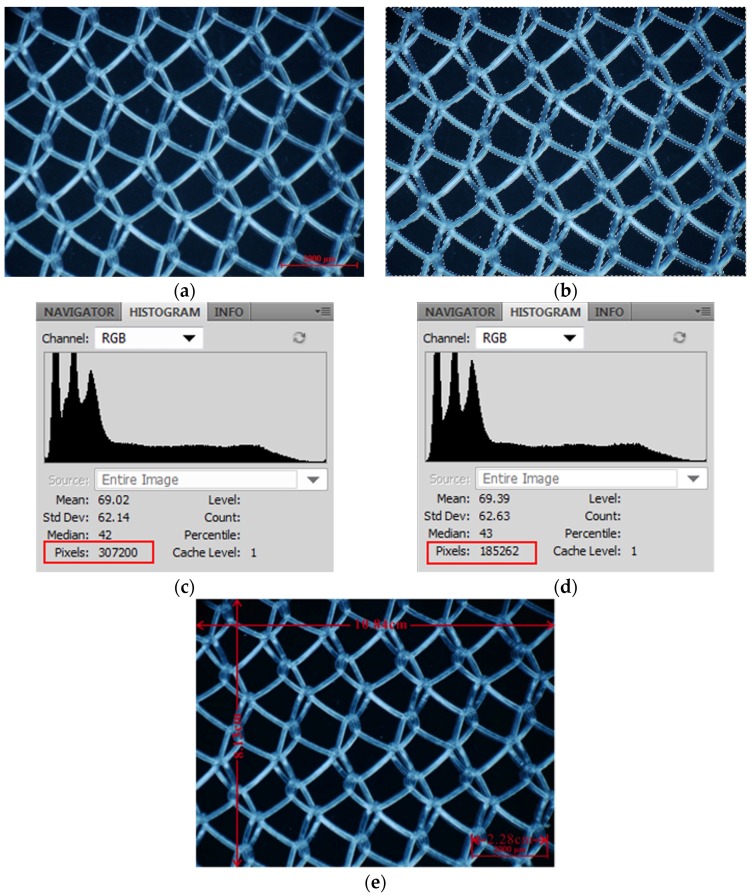
(**a**) Photo at 20× magnification; (**b**) Pore areas of whole picture with Magic Wand Tool; (**c**) Information on the whole picture—the red rectangle values are pixel values of the whole picture; (**d**) Information of all selected pore areas—the red rectangle value is the total pixel value of pore areas; (**e**) Computer screen scale of length and width of the image photographed at 20× magnification with the genuine scale bar at the bottom.

An example is the Bard Polysoft Hernia Patch shown in Figure 7: porosity, calculated by the area method, was 60.3%. Genuine length was 0.951 cm, and genuine width was 0.713 cm, calculated by the ratio of computer screen scale to genuine scale bar length. The genuine area of the portion photographed was 0.678 cm^2^.

### 4.3. Relationship between Prostheses Properties

The diameters of filaments and the stitch densities of all devices were represented in 3D scatter graphs to explore the relationship between structure characteristics, *i.e.*, filament diameter and stitch density (wale and course density). The maximum pore size and porosities of all devices were also represented in 3D scatter graphs. The relationship between thickness and weight per cm^2^ of all devices was represented in histograms. Both 3D scatter graphs and histograms were drawn by OriginPro 8.5 software.

## 5. Conclusions

Warp-knitting techniques are nowadays widely accepted in the manufacture of hernia prostheses because of their sufficient strength and improved elasticity compared to woven fabrics. The structural characteristics of commercial devices with warp knitting structure demonstrated that the filament diameter, stitch density and loop structure play important roles in weight and porosity, which will have an effect on foreign body reaction and rigidity of scar tissue. Warp knitting mesh with thinner filaments and smaller stitch density will possess larger porosity and less weight. The area method to calculate the pores has the advantage of describing the maximum and minimum pore size, as well as pore size distribution, but still lacks capacity in 3D structures. The weight method can describe stereoscopic void space ratio but lack the surface distance between filaments. Further avenues involving Micro CT scan are worth exploring to achieve a more appropriate description of porosity. It was observed that porosity will be distinctly different when taking 1-mm distance into consideration, and devices with larger porosity will not absolutely exhibit larger maximum pore size. Hernia prostheses on the market need more information of their pore characteristics to give clear instruction to surgeons.

From the view of knitting technology, it was concluded that both single-bar and two-guide bar structures can be selected to prepare porous structure fabrics. Two-guide bar structures can form specific net shapes, such as diamonds and hexagons shape, the larger pore size and porosity than single-bar structure. Open-lap morphologies in the same knitting-lapping structure have an impact on effective porosity, maximum pore size and pore size distribution. When choosing a single-guide bar structure, machine gauge, warp run-in and extension in heat-setting finishing should be finely designed to avoid an effective porosity of zero value.

Finally, we suggest that experimental meshes with the same knitting-lapping structures but different stitch densities or the same weight but different knitting-lapping structures can be produced to conduct in-depth study into structures and their impact on mechanical properties. For example, the ratio of wale density to course density probably has an effect on anisotropy regarding the elasticity in longitudinal and horizontal direction under specific force. Some two-guide bar structures with symmetric loops may have no anisotropy in strength or elasticity. The tear strength should also be investigated to verify the lowest stitch densities resistant to the abdominal force. Future studies of the above mechanical properties are planned. Considering the anisotropy of the abdominal wall, the placement direction of a warp knitting mesh is very important to improve abdominal wall compliance after implantation. The detailed description of warp knitting mesh in this article will help surgeons to be more familiar with the structure.
